# The Treatment of Inflammation

**Published:** 1900-05

**Authors:** W. F. Church

**Affiliations:** Chicago, Ill.


### THE TREATMENT OF INFLAMMATION.

BY W. F. CHURCH, M.S., M.D.,

CHICAGO, ILL.

No subject in the domain of therapeutics is so important as this. Under the light of the newer pathology it has assumed even greater prominence than before. To have learned the proper method of treating inflammation, in its different phases and conditions, is to have mastered in a large degree the treatment of disease. The student of to-day is not hampered by a multiplicity of causes as of old, for it is now known that true inflammation, in all instances, is due to the presence and action of germs. As a rule, fever is caused by infection. An exception is made in the healing of an aseptic wound where it is caused by the action of nucleins, fibrin-ferment and products of necrosis. It follows, then, that the treatment of an elevation of temperature, signifying what it may, a felon or pneumonia, is the treatment of an infection. It is not easy to make clear distinctions between local and general infections, for in either the germs must gain admission through some port of entry and the amount of opposition from the cells determines the extent of the invasion. Whether typhoid fever is a local infection with constitutional symptoms or a general infection from the beginning, does not modify the fact that local processes, in some cases abscesses, may occur in the course of the disease. Certain principles apply equally well to an infection through a wound, the tonsils, or any part of the alimentary tract. Because of the wide application of these, it is difficult to tell what lies in the province of surgery or in that of medicine, and, in fact, it makes no difference, as the physician is supposed to be skilled in both. However, to my mind, a knowledge of the proper use of the knife in inflammation is not equal to that required to meet the conditions in other ways.

If the treatment of inflammation were to be limited to the attainment of one object, that object should be to so influence conditions that the cells should be furnished the proper amount of nourishment. The degree of bodily resistance to disease depends on the vitality of the cells. If local conditions be such that nutriment cannot reach them or the blood fails to properly distribute its pabulum, then they cannot offer to germs sufficient resistance. For the physician to assist in furnishing the needed nutrition it is necessary to be familiar with comparative conditions at any given time. If the veins are unable to carry away the blood as rapidly as it is brought to the part by the arteries, stagnation will occur, the lymph channels will become pressed, and nutriment cannot reach the tissue cells, nor phagocytes the germs, which, being walled off, grow and multiply rapidly. There is also an accumulation of toxins and waste nitrogenous material. If the arterial current is too much retarded, toxic material is inadequately swept away, an insufficient number of phagocytes is brought to attack the microbes, and cell nutrition likewise suffers. If too much blood is carried to a part the excess can be remedied in two ways : first, by lessening the propelling force; second, by increasing the venous circulation.

Years ago, Hilton demonstrated the efficacy of rest, not only of the parts affected, but rest of the entire body. It is estimated that the heart-beats are 900 less an hour when lying down than when standing. A reduction of fifteen beats a minute will decidedly lessen the amount of blood carried to a part. A further reduction can be made by the abstention from animal food and the use of a light diet. Additional results may be obtained by recourse to aconite or drugs of a similar action. It cannot be expected that much aid will be given by a few hours' restricted diet, but rest and arterial sedatives will be immediately effective.

Some are in favor of the addition of small doses of digitalis, which slows the heart's action and restricts the caliber of the vessels. If the location be suitable, the venous circulation may be increased by the elevation of the parts, which will at the same time lessen the arterial supply. The use of heat may also help the blood to move onward by producing dilatation of the veins, at the same time aiding nutrition by a similar effect on the lymph channels. When indicated, it is likewise valuable for the relief of pain. Cold constricts the blood-vessels and may relieve pain, but if too long used may destroy the vitality of local cells. Local blood-letting is of value to relieve hyperemia, but cupping may answer the same purpose. Incision, when indicated, should not be too long delayed, as the blood stasis is relieved and germs are swept away, while rest is afforded by the continous drainage.

In cases of inflammation, where the circulation is weak, heart stimulants, like strychnia and digitalis, are needed and a sufficient amount of nitrogenous food. The most careful attention should be paid to the supply of proper nourishment, not only to strengthen the heart, but to maintain the needed number of leucocytes. It is well known that leucocytosis is produced in health after the ingestion of a meal, and the same effect may be expected in disease. Naucrede says, "Blood serum is well known to be germicidal in virtue of the nucleinic acid it contains, dissolved out of, or resulting from, the phagocytic leucocytes." It is claimed there is a definite relation between the two. In health this normal germicidal property answers all purposes, but is unable to withstand an invading host of microbes, hence the need of leucocytosis, which has been shown by laboratory experiments to have increased the bactericidal properties of the blood sixfold. In addition to what may be expected from proper food, leucocytosis may be produced by the administration of neuclein. A one-half per cent. solution of neuclein is fatal to typhoid or cholera bacilli. This action is a good illustration of its germicidal properties in the blood. Nuclein has proved of value in typhoid fever, pneumonia, and many other diseases of microbic origin. It has relieved follicular tonsillitis, in my practice, more quickly than any other remedy. The further claim is made that not only are the white cells increased, but also the red corpuscles and hemoglobin.

In addition to the means that have been enumerated for the conservation of tissue cells and the bactericidal properties of blood serum, there yet remains the valuable service rendered by stimulating the excretory organs. Toxins and pathogenic organisms are swept in the blood stream to different parts of the body, and may find lodgment in such organs as the liver and kidneys. It becomes highly important, then, to increase the function of the emunctories in order that these intruding elements, together with waste nitrogenous material, may be expelled from the body.

Dr. Adami and his assistants have demonstrated that white blood cells, wandering through the intestinal wall in search of pabulum, are, on their return to the tissues, often found to contain bacteria. Benign bacilli that constantly swarm in the intestinal canal may become virulent upon the invasion of the body by another kind. It is of great importance, therefore, to regulate the action of the bowels and administer intestinal antiseptics. The devastating action of germs in inflammation may be met in another way. This is by the administration of a serum that shall be a specific antidote to the poison produced by the pathological processes which they excite. Good results have been obtained in facial erysipelas by the use of antistreptococcic serum, but a positive effect is not realized in every case. There is much to be hoped for in the use of serum, but, with few exceptions, the stage is as yet one of expectancy.

Unguentum Credé is a remedy now on trial which should be mentioned, since its use does not interfere with any other line of treatment. It is recommended in a pure or mixed staphylococcus or streptococcus infection. An application by inunction is made once daily, 45 grains being used at one time. Twenty or thirty minutes are required for this performance. A number of the leading physicians in this city are testing this remedy, the so-called metallic antitoxin, and have obtained good results in some cases, according to reports. The intravenous or subcutaneous injection of normal salt solution should not be omitted from careful consideration in severe cases. It is unnecessary to speak of the disinfection of infected wounds or of the employment of sponge-baths in high temperature. Such measures are expected to be employed whenever indications appear.

It is also unnecessary to dwell on the use of tonics in the chronic form of inflammation or during convalescence.

No attempt has been made to cover the broad field indicated by the subject, but rather to present what seems to the writer the most essential features, which may be briefly summarized as follows:

The support of the vitality of the cell by furnishing proper nutrition. The production and increase of the leucocytes. The necessary surgical treatment. The stimulation of the emunctories. The use of serums and metallic antitoxins.

*1125 W. North Avenue.*

